# Decreasing missingness in race and ethnicity data by inclusion of preferred language for mapping to aggregate categories

**DOI:** 10.1093/haschl/qxaf234

**Published:** 2025-12-05

**Authors:** Zoe Grabinski, Farah Kader, Danielle Bayer, Lan N Ðoàn, Dowin Boatwright, Stella S Yi, Kar-mun Woo

**Affiliations:** Ronald O. Perelman Department of Emergency Medicine, New York University Langone Health, New York, NY 10016, United States; Department of Pediatrics, New York University Langone Health, New York, NY 10016, United States; Department of Population Health, New York University Langone Health, New York, NY 10016, United States; Ronald O. Perelman Department of Emergency Medicine, New York University Langone Health, New York, NY 10016, United States; Department of Population Health, New York University Langone Health, New York, NY 10016, United States; Ronald O. Perelman Department of Emergency Medicine, New York University Langone Health, New York, NY 10016, United States; Department of Population Health, New York University Langone Health, New York, NY 10016, United States; Ronald O. Perelman Department of Emergency Medicine, New York University Langone Health, New York, NY 10016, United States

**Keywords:** health equity, quality and patient safety, race and ethnicity

## Abstract

**Background:**

Accurate and complete patient race and ethnicity data are essential for informing health care quality and patient safety initiatives. However, missing data remain a persistent issue. We aimed to explore the utility of preferred language to impute patient race and ethnicity.

**Methods:**

This was a retrospective analysis from 3 emergency departments in New York City, from June 1, 2023, to May 31, 2024. We leveraged a mapping schema for imputation of missing race and ethnicity data using preferred language for categorization into the Office of Management and Budget’s 7 categories. We examined concordance between preferred language and predicted categories.

**Results:**

The proportion of patients with missing race and ethnicity data decreased from 9.7% to 8.6%, reducing missingness by 11.1%. The greatest proportion of change with the use of preferred language was for Middle Eastern and North African patients (14.7%).

**Conclusion:**

Our findings support that language-based imputation has the potential to reduce missing race and ethnicity data and may be a helpful tool in quality improvement and research efforts. For health systems where race and ethnicity fields may not be fully detailed or have a high rate of missing data, the use of language may serve as a valuable adjunct in improving the comprehensive picture of a population.

## Introduction

Accurate sociodemographic data are necessary to inform the development of health care quality and safety initiatives to address health disparities.^1^ However, the accuracy and completeness of such data, particularly patient race and ethnicity, are a persistent issue, with detailed ethnic group data only available for approximately one-third of patients.^2^ Nonrandom missing race and ethnicity data can introduce bias, obscure differences across groups facing distinct health challenges, and decrease statistical power in health research.^3,4^ In the absence of complete self-reported data, imputation models can be critical tools for population-level analyses. This study explores the utility of patients’ preferred language to impute missing race or ethnicity.

## Methods

In 2024, the US Office of Management and Budget (OMB) updated its’ prior 1997 recommendations for the categorization of federal data on race and ethnicity. Within the next few years, federal reporting agencies will be expected to comply with the future incorporation of now 7 minimum race and ethnicity categories: American Indian or Alaska Native (AI/AN), Asian, Black or African American, Hispanic or Latino, Middle Eastern and North African (MENA), Native Hawaiian and Pacific Islander (NH/PI), White, and Other. Prior to the OMB's recommendation to combine race and ethnicity categories into 1 variable and to add the MENA category, our department explored using preferred language for imputation of race and ethnicity exclusively when race and ethnicity data were otherwise incomplete. This approach reduced missing race and ethnicity data, defined as patients with “Unknown,” “Refused,” or “Other” race, from 30% to 10% (67% decrease).^5^ This schema approach has since undergone iterative changes, accounting for the updated OMB recommendations, additional race and ethnicity categories since added to our institutional electronic health record (including MENA race and non-Hispanic ethnicities), and patient-centered qualitative analyses.^6,7^

We analyzed retrospective patient data from 3 emergency departments (EDs) in a New York City (NYC) academic health network, collectively treating over 300 000 patients annually. At the time of ED registration, patients are asked to confirm race, ethnicity, and preferred language, which are recorded in an integrated electronic health record (Epic Systems Corporation, Verona, WI). We included patients managed in the ED from June 1, 2023, to May 31, 2024. Proportions reported are on a per-patient basis.

Table 1 displays the race, ethnicity, and language (REaL) schema mapping to OMB categories, developed via consensus opinion by experts in patient quality and safety, equity, and population health.^8,9^ The mapping also incorporates the published REaL literature and considers NYC demographics and US Census data.^10^  ^,11^

**Table 1.** Self-reported race, ethnicity, and language mapping schema to an OMB category.

OMB category^a^	Race^a,b^	Ethnicity^b^	Language^c^
AI/AN	American Indian or Native or First Nations or Indigenous Peoples of the Americas or Alaska Native	Apache, Cherokee, Iroquois or Haudenosaunee, Central American Indian, Mexican American Indian (eg, Mixteco or Nahua or Otomi or Tlapaneco), Southern American Indian (eg, Quechua or Kichwaor Shuar or Aymara)	None
Asian	Asian	Bangladeshi, Chinese, Filipino, Indian (Asian), Japanese, Korean, Vietnamese	Bengali, Burmese; Chinese—Cantonese; Chinese—Fuzhounese/Fuzhou; Chinese—Mandarin; Chinese—Taiwanese/Fukinese; Chinese—Toishanese/Toishan, Hindi, Japanese, Kannada, Korean, Laotian, Malayalam, Nepali, Punjabi, Tagalog-Filipino, Thai, Urdu, Uzbek, Vietnamese
Black or African American	Black or African American	African American, Black, Haitian, Jamaican, Nigerian, Trinidadian	Bambara, Creole-Haitian/Haitian Creole/French Creole, Fulani, Ibo, Somalian, Swahili, Twi, Wolof, Yoruba
Hispanic or Latino	Hispanic or Latino or Spanish	Andalusian, Argentinean, Asturian, Belearic Islander, Bolivian, Canal Zone, Canarian, Castillian, Catalonian, Central American Indian, Chicano, Chilean, Columbian, Costa Rican, Criollo, Cuban, Dominican, Ecuadorian, Gallego, Guatemalan, Honduran, La Raza, Latin American, Mexican, Mexican American Indian (eg, Mixteco or Nahua or Otomi or Tlapaneco), Nicaraguan, Panamanian, Paraguayan, Peruvian, Puerto Rican, Salvadoran, South American Indian (eg, Quechua or Kichwa or Shuar or Aymara), Uruguayan, Valencian, Venezuelan	Spanish
MENA	Middle Eastern or Northern African	Armenian, Egyptian, Iranian, Turkish, Yemeni	Arabic, Armenian, Farsi-Persian, Turkish
NH/IPI	Native Hawaiian, Pacific Islander	Samoan, Tongan, Guamanian or Chamorro	Marshallese
Other	Not listed	Not listed, I don't identify as a more specific racial or ethnic group	None
Unknown or Refused	Prefer not to answer, Don't know	Prefer not to answer	None
White	White	Irish, Italian, Polish, Romanian, German	Albanian, Bulgarian, Croatian, European Portuguese, Georgian, German, Greek, Hungarian, Italian, Latvian, Polish, Romanian, Russian, Serbian, Ukranian, Yiddish
Unmapped^d^	Not applicable	Guyanese, Israeli, Jewish	ASL, English, French, Hebrew, Portuguese

^a^Race and ethnicity are mapped equivalently to an OMB category. If a patient self-selects a race and ethnicity that mapped to more than 1 OMB category, they were added to each of the OMB categories that were self-reported. This approach was chosen to not obscure racial and ethnic diversity of a population by creating a separate multiracial group.

^b^Concordance was defined as any patient who self-reported the mapped OMB category as 1 of their selected racial or ethnic categories.

^c^Languages as displayed in the most updated self-reported fields for race in the electronic health record. Only those languages for which the number of patients in the population ≥10 are displayed.

^d^These ethnicities and languages remain unmapped to any OMB category based on NYC demographic characteristics and known multi-race or multi-ethnic characteristics of the population.

Abbreviations: AI/AN, American Indian/Alaska Native; ASL, American Sign Language; MENA, Middle Eastern or North African; NH/IPI, Native Hawaiian or Pacific Islander; NYC, New York City; OMB, Office of Management and Budget.

As the first step to using language as a proxy for race and ethnicity, we calculated concordance between patients’ primary language and selection of a race and ethnicity category that corresponded to 1 of the “predicted” races and ethnicities for that language (Table 1). We limited imputation to languages selected by 60 or more patients and that had a concordance of 75% or greater to the predicted OMB category. These thresholds were determined through sensitivity analysis examining sample sizes and concordance percentages for a 95% CI within ±10% (62.1%–85.3%).

Second, we imputed OMB categories for those patients with “Unknown,” “Refused,” or “Other” race and ethnicity but had self-reported language. For languages with 60 or more patients, we also explored the proportion of patients with the highest rates of missing race and ethnicity responses. This investigation adhered to the New York University Grossman School of Medicine process for quality improvement (QI) activities and met criteria for QI designation.

## Results

Table 2 presents the concordance analysis results. Languages with *n* ≥ 60 patients were used for imputation, except for Turkish and Uzbek, which had language-to-race concordance rates of 63.0% and 13.9%, respectively. All other languages had concordance with the mapping schema (Table 1) exceeding 95%, except for Albanian (94.4%), Bengali (91.1%), and Urdu (84.4%).

**Table 2.** Concordance of self-reported language with OMB category.

Language (*n*)^a^	Mapped OMB category	Concordance,^b,c^ *n* (%)
Spanish (21 803)	Hispanic or Latino	21 606 (99.1)
Russian (2202)	White	2122 (96.4)
Chinese—Cantonese (2087)	Asian	2087 (100)
Chinese—Mandarin (2078)	Asian	2072 (99.7)
Arabic (1267)	MENA	961 (75.9)
Polish (418)	White	418 (100)
Bengali (291)	Asian	265 (91.1)
Chinese—Fuzhounese/Fuzhou (214)	Asian	213 (99.5)
Italian (207)	White	203 (98.1)
Albanian (143)	White	135 (94.4)
Creole—Haitian/Haitian or French Creole (141)	Black	139 (98.6)
Georgian (128)	White	123 (96.1)
Greek (128)	White	124 (96.9)
Urdu (128)	Asian	108 (84.4)
Korean (104)	Asian	104 (100)
Chinese—Toishanese/Toishan (93)	Asian	93 (100)
Turkish (92)	MENA	58 (63.0)
Ukranian (72)	White	71 (98.6)
Yiddish (71)	White	70 (98.6)
Japanese (65)	Asian	65 (100)
Uzbek (65)	Asian	9 (13.9)
Vietnamese (65)	Asian	64 (98)

^a^Total number of patients who reported the language within the population and reported a race and/or ethnicity. Only languages with *n* ≥ 60 are displayed.

^b^Patients who self-reported the mapped OMB category.

^c^Patients are counted if at least 1 race or ethnicity selection maps to the OMB category.

Abbreviations: MENA, Middle Eastern and North African; OMB, Office of Management and Budget.

Table 3 displays the imputation results using language mapping. The proportion of patients with missing race and ethnicity data decreased by 11.1%. The greatest proportion of change within OMB categories was for MENA patients (14.7%).

**Table 3.** Proportion of patients in OMB categories following mapping schema using language.

OMB category^a^ (*n* = 190 602)	Patients within OMB category using self-reported race and ethnicity, *n* (%)	Patients within OMB category following addition of language mapping, *n* (%)	Delta (%)
AI/AN	3870 (2.0)	3870 (2.0)	0
Asian	16 006 (8.4)	16 314 (8.6)	1.9
Black	28 840 (15.1)	28 856 (15.1)	0.1
Hispanic or Latino	49 706 (26.1)	50 609 (26.6)	1.8
MENA	3791 (2.0)	4349 (2.3)	14.7
NH/PI	630 (0.3)	630 (0.3)	0
White	69 321 (36.4)	69 604 (36.5)	0.4
Other/Unknown/Refused	18 418 (9.7)	16 370 (8.6)	−11.1

^a^Patient counted if at least 1 race or ethnicity selection maps to the OMB category in the mapping schema of Table 1. Multiracial patients are counted once in each category.

Abbreviations: AI/AN, American Indian or Alaska Native; MENA, Middle Eastern and North African; NH/PI, Native Hawaiian and Pacific Islander; OMB, Office of Management and Budget.

Table 4 highlights the proportion of missing race and ethnicity data by languages with the highest missing race and ethnicity data (>25% of the languages) that could be mapped to an OMB category. These languages were Arabic (30.0%), Portuguese (29.6%), and Uzbek (26.1%). Uzbek was not mapped due to poor language-to-race concordance (13.9%) within our population.

**Table 4.** Missing self-reported race and ethnicity data within language groups.

Language^a^ (*n*)	Patients with missing race and ethnicity, *n* (%)
English (140 929)	14 804 (10.5)
Spanish (22 698)	895 (3.9)
Russian (2366)	164 (6.9)
Chinese—Mandarin (2173)	95 (4.4)
Chinese—Cantonese (2165)	78 (3.6)
Arabic (1811)	544 (30.0)
Polish (439)	21 (4.8)
Bengali (356)	65 (18.3)
French (348)	67 (19.3)
Italian (225)	18 (8.0)
Chinese—Fuzhounese/Fuzhou (224)	10 (4.5)
Albanian (174)	31 (17.8)
Urdu (163)	35 (21.5)
Creole—Haitian/Haitian or French Creole (157)	16 (10.2)
Georgian (151)	23 (15.2)
Greek (137)	9 (6.6)
Turkish (118)	26 (7.1)
Korean (112)	8 (12.1)
Hebrew (99)	12 (12.1)
Chinese—Toishanese/Toishan (98)	5 (5.1)
American Sign Language (ASL) (94)	13 (13.8)
Portuguese (88)	26 (29.6)
Uzbek (88)	23 (26.1)
Ukranian (80)	8 (10.0)
Yiddish (79)	8 (10.1)
Japanese (71)	6 (8.5)
Vietnamese (70)	5 (7.10)

^a^Total number of patients who reported the language within the population. Only languages with *n* ≥ 60 are displayed.

## Discussion

Our language-to-OMB category mapping schema decreased the proportion of patients with missing race and ethnicity data to 8.6% missingness, a significantly lower rate than the up to 30% reported in other health care studies.^1,2,5^ The findings indicate that this mapping schema has potential for research or QI efforts, particularly benefitting patients prone to misclassification in electronic health records. While language-to-race mapping showed the greatest increase in identifying MENA patients, concordance calculations varied among MENA-associated languages such as Arabic and Turkish and were generally lower than other OMB groups. This can be attributed to several factors, including the previous categorization of MENA in the White OMB category prior to 2024, racial diversity among descendants of the MENA region, and varying self-perceptions of race.^4,12^ Conversely, Spanish-speaking patients were highly concordant to the Hispanic or Latino OMB category, as were speakers of specific Asian languages (eg, Cantonese, Mandarin) to the Asian OMB category. For both Spanish and Asian language groups, imputation using language mapping resulted in modest decreases in missing race data; Asian-language speakers may be able to be mapped to specific Asian groups, although more research is needed.

The performance of the proposed imputation method is dependent on understanding the racial and ethnic diversity of the patient population, considering regional sensitivities and adapting to the local context.^13^ This approach may be limited for English-preferring or multilingual patients, including multigenerational Americans across race and ethnicity groups. For these patients, their preferred language may not provide meaningful information about race or ethnicity. Validation strategies, such as surname algorithms, neighborhood-level data, and other methods, may improve completeness for these groups.^14-19^

A limitation is the inability to confirm whether the recorded race, ethnicity, or preferred language captured are truly self-reported, the gold standard, as some may have been inadvertently entered based on staff observation or data carried over as a default and reconfirmed from prior visits. Low report of MENA race may reflect limited familiarity with the newly introduced category, rather than lack of patient identification. As institutions transition to incorporating updated OMB race and ethnicity standards, language-based imputation may serve as a temporary adjunct to support more complete data capture until self-reporting processes become more consistent. These limitations may lead to underestimation of the true impact of language mapping or even differential misclassification.^20^ However, calculating concordance as a first step provided empirical justification for using language as a proxy for race and ethnicity under certain conditions and minimized the risk of misclassification. Preferred language may be particularly impactful for reducing missingness in health systems where detailed race and ethnicity options are not available for selection by the patient—for example, if an electronic health record has not yet included MENA as 1 of its race categories. Our previous iteration reduced race and ethnicity missingness from approximately 30% to 10% (66.7% decrease).^4^

To improve upon this method, future research should focus on validating the mapping of language-to-race categories through patient-based surveys and community focus groups in a broad array of communities, but with particular attention to the MENA population. A combined approach for validation should aim for broad patient reach while also allowing community expertise to refine processes.

While language-imputed race and ethnicity should not be used on an individual level, such as in clinical settings, we hope that future applications of this method may support department-wide QI, equity efforts, and research projects on a population level. These initiatives are often limited by high rates of missingness in the race and ethnicity data and where there are anticipated lags in the health data infrastructure for race and ethnicity reporting for the next few years.

## Conclusion

While further investigation is needed, our findings support the potential for language-based imputation to reduce missingness in race and ethnicity responses. To improve quality of care for patients this is a promising approach to improve data comprehensiveness.^9^ Importantly, language should not be viewed as a substitute for self-reported race and ethnicity but rather as a contextual variable that can partially reduce missingness in population-level analyses. Even with language-based imputation, race and ethnicity data (ie, patient diversity) will likely remain underestimated, underscoring the continued importance of investing in accurate and patient-centered self-report processes. Nonetheless, this method can improve providers’ and researchers’ understanding of their patient populations, ultimately leading to informed and fair health care practices.

## Supplementary Material

qxaf234_Supplementary_Data
